# Exploring the role of anthropometric measurements to assess nutritional status in amyotrophic lateral sclerosis: a longitudinal prospective cohort study

**DOI:** 10.1080/21678421.2024.2434176

**Published:** 2024-12-16

**Authors:** Sarah Roscoe, Scott P. Allen, Christopher McDermott, Theocharis Stavroulakis

**Affiliations:** Division of Neuroscience, School of Medicine and Population Health, Sheffield Institute for Translational Neuroscience, University of Sheffield, Sheffield, UK

**Keywords:** Amyotrophic lateral sclerosis, anthropometry, body composition, nutritional assessment, malnutrition

## Abstract

**Objective:**

To observe longitudinal correlations between limb anthropometry against weight, BMI and functional decline in patients with amyotrophic lateral sclerosis.

**Methods:**

A longitudinal, prospective, cohort study was undertaken. Four consecutive measurements of weight, height, triceps skinfold thickness (TSF), mid-upper arm (MUAC) and calf circumferences were collected at three-monthly intervals. Fat- and lean body mass were estimated using measurements of TSF and derivations of arm muscle area, respectively. Correlation analyses indicated associations between anthropometric assessments and functional decline (ALSFRS-R). Longitudinal changes were assessed using repeated measures analyses.

**Results:**

Data from 18 participants was analyzed. At enrollment, weight positively correlated with MUAC (n = 17, p = 0.0001), arm muscle area (n = 17, p = 0.04) and calf circumference (n = 17, p < 0.0001). The ALSFRS-R score negatively correlated with weight (n = 17, p = 0.03), MUAC (n = 18, p = 0.01), TSF (n = 18, p = 0.04), and calf circumference (n = 18, p = 0.003). Function significantly declined by a difference of 6.3 points per month (p = 0.009). A positive correlation was observed between the changes in weight and calf circumference over nine months (r = 0.70, p = 0.02, *n* = 10).

**Conclusion:**

Limb anthropometric measurements may be surrogate indicators of weight and BMI; TSF may be a practical, reliable indicator of fat mass, whilst changes in calf circumference may be alternatively used to monitor changes in nutritional status in the clinic.

## Introduction

The prognosis of an individual living with amyotrophic lateral sclerosis (ALS) is often associated with their nutritional status (1), with predicted survival depending on the method and time of nutritional assessment (2). However, nutritional screening in ALS lacks standardization; whilst a number of approaches and international guidelines exist, these are often not supported by a strong evidence base (2–7).

Patients with ALS may experience asymmetric muscle wasting as a direct result of continued denervation, with the potential for irreversible malnutrition-associated lean body mass (LBM) and fat mass (FM) loss occurring as a result of prolonged energy-protein intake imbalance (8). Malnutrition is estimated to affect 16-55% of people living with ALS and is associated with a 3.5-fold increased risk of death (1,9). Denervation and malnutrition both contribute to changes in weight and BMI (1,10).

In a UK survey of 130 dietitians, 92% reported the use of body weight and BMI to assess nutritional status, with 82% reporting the calculation of percentage weight change over 3-6 months (11). However, accurate measurements are not always practical to obtain, especially from non-ambulatory patients (12): almost two-thirds of UK dietitians reported access to wheelchair scales, with approximately half having access to hoist scales (11). UK clinical guidelines do not recommend BMI as an assessment of nutritional status in patients with ALS to prevent the necessity of height measurements (3). Moreover, weight measurements do not detail the proportion or anatomic distribution of FM and LBM (13,14), which is known to vary with disease progression, age, sex and ethnicity (15,16). Longitudinal changes in body composition should be assessed alongside to better monitor disease progression (17,18).

### Limb anthropometric assessments as indicators of body composition

Anthropometric measurements such as triceps skinfold thickness (TSF), mid-upper arm circumference (MUAC) and calf circumference (CC) are cost-effective, clinically-accessible indirect proxies of body composition (19). MUAC and CC measurements encompass bone, muscle and subcutaneous fat, and do not distinguish between FM and LBM (20–22). MUAC and CC have been proposed as surrogate indicators of BMI in healthy (23–25), sarcopenic (26,27) and aging populations (28). TSF can be used as proxy estimates of localized, subcutaneous FM (29). TSF can be used in combination with MUAC to derive the arm muscle area (AMA), an index of upper-arm LBM (20,30).

Reference of these indices to standardized thresholds, or percentiles, developed in healthy cohort validation studies could be used to indicate malnutrition (31,32). However, no clinically-significant thresholds exist for limb anthropometric measurements to indicate nutritional status or malnutrition in ALS. Regardless, available evidence from the UK and Canada indicates 9-31% of dietitians use MUAC measurements in their day-to-day practice with ALS patients (11,33).

Our study aims to enhance our understanding of the role of limb anthropometric measurements in assessing nutritional status and identifying malnutrition in patients with ALS.

## Materials and methods

### Study design

This was a single-site, longitudinal, observational, prospective study of patients with a confirmed diagnosis of ALS, recruited between October 2021 and August 2022. Assessments were undertaken at three-monthly intervals at month 0 (M0) (enrollment), M3, M6 and M9. Exclusion criteria were limited to an underlying, unmanaged co-morbidity, or a decision-making incapacity preventing informed consent. Favorable opinion was obtained from the London-Fulham NHS Research Ethics Committee (21/PR/0092).

### Assessment of disease severity and progression

Disease severity was assessed using the self-administered ALSFRS-R (34) and the King’s College staging system (35). The rate of disease progression (ΔALSFRS-R) was calculated by: (48 - ALSFRS-R total score at time of assessment)/disease duration from symptoms onset (months). Disease duration was defined as the interval between participant-reported date of first MND symptom onset and the first study visit (M0), in months. The ΔALSFRS-R was calculated to categorize participants into slow (<1.1 point/month) and fast progressors (≥1.1 point/month) (36).

### Assessment of nutritional status

Body weight to the nearest 0.1 kg (SECA 875) and height to the nearest 0.1 cm (SECA 213) were recorded in light clothing and shoes in an unaided standing position. Percentage weight change was calculated compared to participant weight before symptom onset or diagnosis. BMI (kg/m^2^) was calculated and categorized according to the World Health Organization classification (37). MUAC was measured to the nearest 0.1 cm using a non-elasticated anthropometric measuring tape (SECA 201) (32,38). TSF was measured to the nearest 0.2 mm in triplicate using a Skinfold Caliper (Harpenden) (38). MUAC and TSF were measured symmetrically at the mid-point between the acromion and olecranon processes. AMA (cm^2^) was calculated by: [MUAC (cm) – (TSF (cm) x π)]^2^/(4 x π) (30). Calf circumference to the nearest 0.1 cm was measured symmetrically at the largest part of the calf in a relaxed seated position at an angle of 90° (SECA 201). All anthropometric measurements were conducted by the same researcher. The MUAC, CC and TSF were compared with published standard percentile tables for age and sex (31,32,39). The percentage of body fat (%FM) was estimated using the equation by Tandan et al. (2022)^1^ (40). FM (kg) and LBM (kg) were calculated from the computed %FM^2^.

Participant-reported dietary intake was recorded using Intake24, an online 24-hour dietary recall questionnaire (41). Energy intake was compared to the estimated average requirement for the UK population according to age and sex (42).

A risk of malnutrition was indicated when any two of the criteria outlined in Table 1 were met at any time (47).

**Table 1.** Criteria for the identification of the risk of developing malnutrition. Two or more nutritional assessments below the defined thresholds indicated a risk of malnutrition.

Nutritional assessment	Age	Threshold for malnutrition		Reference
BMI (kg/m^2^)	<70 years ≥70 years	≤20kg/m^2^ ≤22 kg/m^2^		(43)
Percentage weight loss from initial body weight (%)		≥−10%		(44–46)
		**Male**	**Female**	
MUAC (cm)	30-39	29.2	25.1	(32)
	40-49	29.2	25.7	
	50-59	28.0	25.1	
	60-69	27.6	25.0	
	70-79	26.7	24.9	
	80+	25.4	23.0	
CC (cm)	30-39	34.1	32.2	(39)
	40-49	34.7	32.8	
	50-59	33.7	32.3	
	60-69	33.4	31.5	
	70-79	32.3	31.1	
	80+	31.1	29.8	
TSF (mm)	30-39	5.9	12.1	(31)
	40-49	6.3	11.5	
	50-59	7.0	12.9	
	60-69	7.2	13.0	
	70-79	7.6	11.5	
	80+	7.0	9.4	
Recommended energy intake (kcal/day)	≤ 64	2500	2000	(42)
	65-74	2342	1912	
	≥ 75	2294	1840	

BMI: body mass index; CC: calf circumference; MUAC: mid-upper arm circumference; TSF: triceps skinfold thickness.

### Statistical analysis

Statistical analysis was conducted using IBM^®^ SPSS^®^ Statistics (IMB SPSS statistics for Macintosh, Version 29.0.1.1) and GraphPad Prism (GraphPad Software Inc, La Jolla, CA, USA, Version 9.3.1). Continuous variables were presented as mean [standard deviation (SD)] or median [interquartile range (IQR)]. Normality was assessed using the Shapiro–Wilk test. Pearson’s or Spearman’s bivariate correlation analysis was plotted with a linear regression line and 95% confidence intervals from the mean. Where the classification of participants into groups according to pre-defined thresholds resulted in small group sizes, the median split technique to create a ‘low’ and a ‘high’ group was utilized for continuous variables; mean values were compared using the Mann-Whitney U Test (48).

Intra-evaluator variability for triplicate TSF measurements was assessed by the relative technical error of measurement (TEM), with acceptability defined as < 7.5% (49). The mean [SD] value of right and left limb measurements was calculated at all time points and compared using paired Samples *t* Tests to identify any significant asymmetrical changes in body composition.

Participant age at M0 was used for all equations and longitudinal analysis. Longitudinal data for all individuals at all time points was analyzed using Dunnett’s mixed-effects (50) or Wilcoxon analyses (51). The change in anthropometric and clinical parameters between month 0 and month 9 was examined for participants who completed all four study visits. Mean values were compared using paired samples t tests or Wilcoxon tests for non-parametric data. Statistical significance was set at *p* < 0.05.

## Results

Twenty-four patients living with motor neuron disease were recruited to this study; recruitment was not restricted by phenotype. Only patients with ALS or a PMA variant of ALS were included in these analyses (*n* = 18 at M0, 16 at M3, 13 at M6 and 10 at M9). Longitudinal demographic, clinical and nutritional assessment values are shown in Table 2. The intra-evaluator relative TEM for triplicate TSF measurements at all time points were acceptable (range: 4.0%-7.1%). No significant differences were observed between right- and left-hand side measurements at any time point (Table S1). Combined mean [SD] values for symmetrical limb measurements are presented.

**Table 2.** Longitudinal demographic, clinical and nutritional assessments. The number of participants per assessment is presented as n/N (percentage of population, %). Continuous data is presented as mean (SD). Median (IQR) is presented for heavily skewed data. Changes in longitudinal data were analyzed for significance using Dunnett’s mixed method for multiple comparisons test for normally distributed data or Wilcoxon matched-pairs signed rank test for non-normally distributed data. Significance observed at *p* < 0.05, highlighted in bold. ALSFRS-R: Amyotrophic Lateral Sclerosis functional rating scale – revised; IQR: inter-quartile range; kcal/day: kilocalories per day; M0-9: Months 0-9; SD: standard deviation; ΔALSFRS-R: change in functional score.

					*P* value		
	M0	M3	M6	M9	M0-M3	M0-M6	M0-M9
n/N	18/24	16/24	13/24	10/24			
**Sex, Male:Female**	16:2	14:2	13:0	10:0			
**Age, years**	62.06 (10.7)						
**ALS phenotype**							
ALS	16/18 (18.9)						
PMA	2/18 (11.1)						
**Site of onset**							
Bulbar	4/18 (22.2)						
Upper limb	5/18 (33.3)						
Lower limb	5/18 (33.3)						
respiratory	2/18 (11.1)						
mixed	2/18 (11.1)						
**Disease duration (months)**	41.50 (42.39) 25.50 (18.50-49.50)	36.63 (22.90) 28.50 (20.50-45.00)	43.15 (24.15) 34.00 (25.00-60.50)	50.50 (26.25) 41.00 (28.00-75.75)			
**Disease Severity**							
**King’s Staging**							
Stage 1	2/18 (11.1)	2/16 (12.5)	1/13 (7.7)	2/10 (20.0)			
Stage 2	5/18 (27.8)	3/16 (18.75)	3/13 (23.1)	–			
Stage 3	3/18 (16.7)	3/16 (18.75)	3/13(23.1)	5/10 (50.0)			
Stage 4	8/18 (44.4)	8/16 (50.0)	6/13 (46.2)	4/10 (40.0)			
**ALSFRS-R (/48)**	32.22 (5.63)	30.94 (6.65)	26.92 (8.78)	26.00 (9.20)	n = 16 0.73	n = 13 **0.008**	n = 10 **0.02**
Bulbar subscale	9.65 (2.73)	9.25 (3.08)	9.00 (3.46)	8.20 (4.13)	n = 16 0.81	n = 13 0.25	n = 10 0.06
Fine motor subscale	7.33 (2.47)	6.63 (3.18)	5.00 (3.37)	4.80 (3.33)	n = 16 0.34	n = 13 **0.003**	n = 10 **0.03**
Gross motor subscale	7.05 (2.41)	7.13 (2.78)	5.46 (2.07)	4.90 (2.13)	n = 16 0.44	n = 13 **0.001**	n = 10 **0.03**
Respiratory subscale	8.28 (3.82)	7.94 (3.80)	7.46 (4.27)	8.10 (3.99)	n = 16 0.74	n = 13 0.19	n = 10 0.28
**ΔALSFRS-R**	0.69 (0.51)	0.62 (0.39)	0.29 (0.47)	0.60 (0.45)	n = 16 0.20	n = 13 0.75	n = 10 >0.99
**Gastrostomy**							
Present	5/18 (27.8)	6/16 (37.5)	6/13 (46.2)	5/10 (50)			
Not present	13/18 (72.2)	10/16 (62.5)	7/13 (53.8)	5/10 (50)			
**Non-invasive ventilation**							
No respiratory support	11/18 (61.1)	8/16	7/13	6/10			
Intermittent use	0/18 (0.0)	2/16	1/13	1/10			
Overnight	6/18 (33.3)	5/16	3/13	2/10			
24-hour use	1/18 (5.6)	1/16	2/13	1/10			
**Anthropometric measurement or indices**							
Weight, kg	n/N = 17/18 79.36 (18.41)	n/N = 16/16 80.18 (18.90)	n/N = 13/13 83.33 (17.86)	n/N = 10/10 83.06 (16.27)	n = 16 0.99	n = 13 >0.99	n = 10 0.57
Percentage weight change, %	n/N = 15/18 −4.40 (7.30)	n/N = 14/16 −4.35 (7.36)	n/N = 11/13 −2.42 (9.61)	n/N = 8/10 −3.52 (10.64)	n = 14 0.99	n = 11 >0.99	n = 8 0.84
Body mass index, kg/m^2^	n/N = 17/18 25.92 (4.43)	n/N = 16/16 26.02 (4.46)	n/N = 13/13 26.01 (4.63)	n/N = 10/10 25.77 (3.81)	n = 16 0.97	n = 13 0.92	n = 10 0.61
Mid-upper arm circumference, cm	n/N = 18/18 29.46 (3.59)	n/N = 16/16 28.80 (3.64)	n/N = 12/13 29.03 (4.17)	n/N = 10/10 28.78 (4.02)	n = 16 **0.047**	n = 12 0.67	n = 10 0.66
Triceps skinfold thickness, mm	n/N = 18/18 13.70 (6.51)	n/N = 16/16 12.70 (5.17)	n/N = 12/13 13.03 (5.50)	n/N = 10/10 12.96 (4.80)	n = 16 0.93	n = 12 0.99	n = 10 0.98
Arm Muscle Area, cm^2^	n/N = 18/18 51.25 (13.71)	n/N = 16/16 49.07 (10.60)	n/N = 12/13 50.14 (11.21)	n/N = 10/10 49.35 (12.01)	n = 16 0.94	n = 12 0.32	n = 10 0.30
Calf circumference, cm	n/N = 18/18 37.22 (3.45)	n/N = 16/16 37.45 (3.49)	n/N = 12/13 38.03 (3.40)	n/N = 10/10 37.99 (3.01)	n = 16 0.81	n = 12 0.98	n = 10 >0.99
**Reported 24hr dietary intake**							
Energy, kcal/day	n/N = 18/18 2238.81 (755.61)	n/N = 16/16 2100.33 (736.53)	n/N = 13/13 2689.27 (1391.13)	n/N = 10/10 2314.23 (906.02)	n = 16 0.71	n = 12 0.89	n = 10 >0.99

### The relationship between assessments of nutritional status, disease severity and rate of disease progression

Relationships between nutritional status and disease severity at M0 and M9 are presented in Figure 1. At M0, significant moderate-to-strong positive correlations were observed between weight and MUAC, AMA or CC, with the strongest relationship observed between weight and CC (*r* = 0.93, *p* = <0.0001, *n* = 17) (Figure 1A). Significant, positive relationships were observed between all limb anthropometric assessments against weight and BMI at M9 (Figure 1B).

Figure 1.Correlation of nutritional assessment against disease severity at each study visit. A) Month 0; B) Month 9. Correlation analysis was conducted using Spearman’s correlation analyses for non-normally distributed data. Significance observed at *p* < 0.05. Significant results highlighted in bold. AMA: arm muscle area; ALSFRS-R: Amyotrophic Lateral Sclerosis functional rating scale – revised; BMI: body mass index; IQR: inter-quartile range; kcal/day: kilocalories per day; M0/9: Months 0/9; MUAC: mid-upper arm circumference; SD: standard deviation; TSF: triceps skinfold thickness; ΔALSFRS-R: change in functional score.

**Figure:**
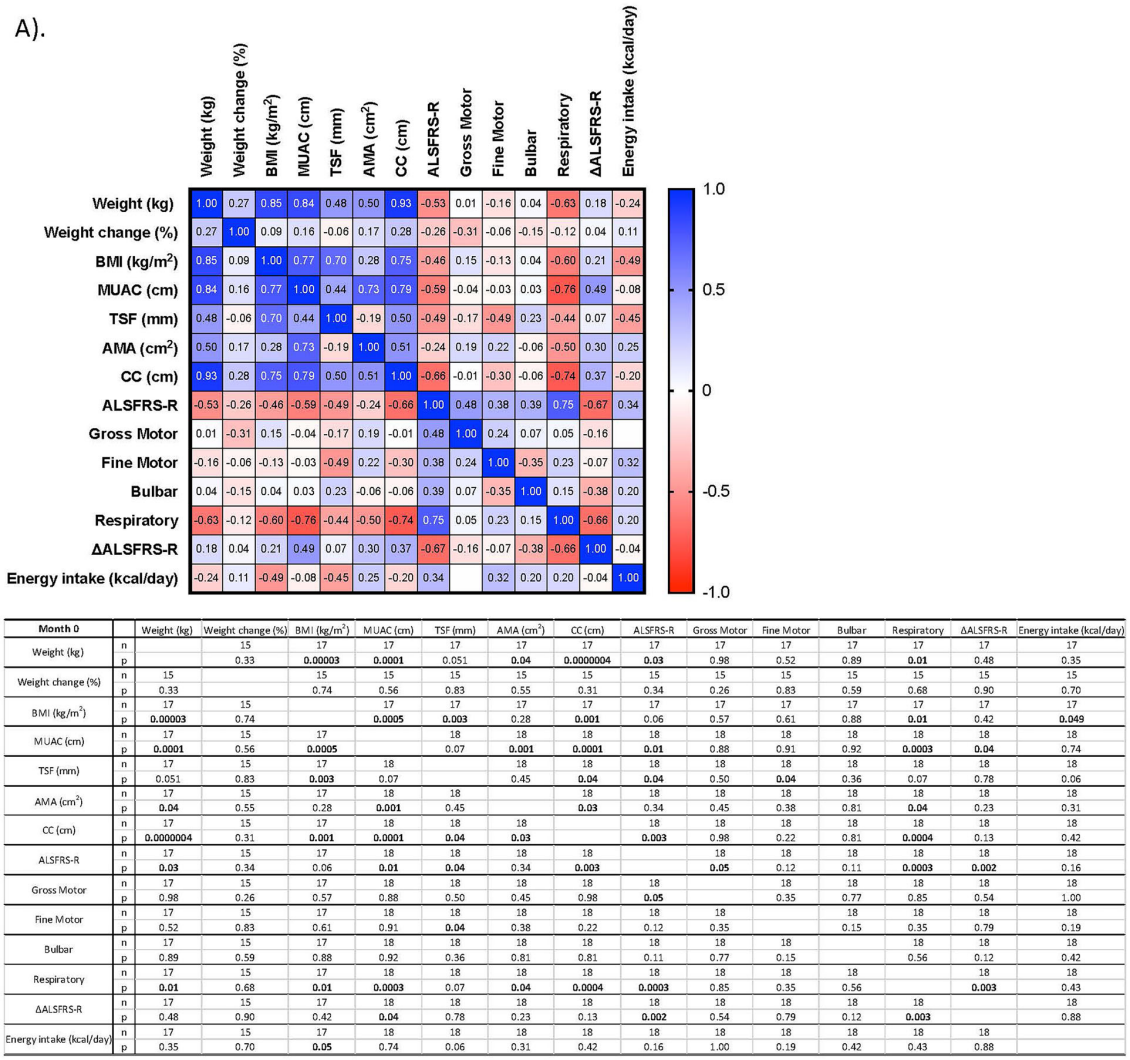

**Figure:**
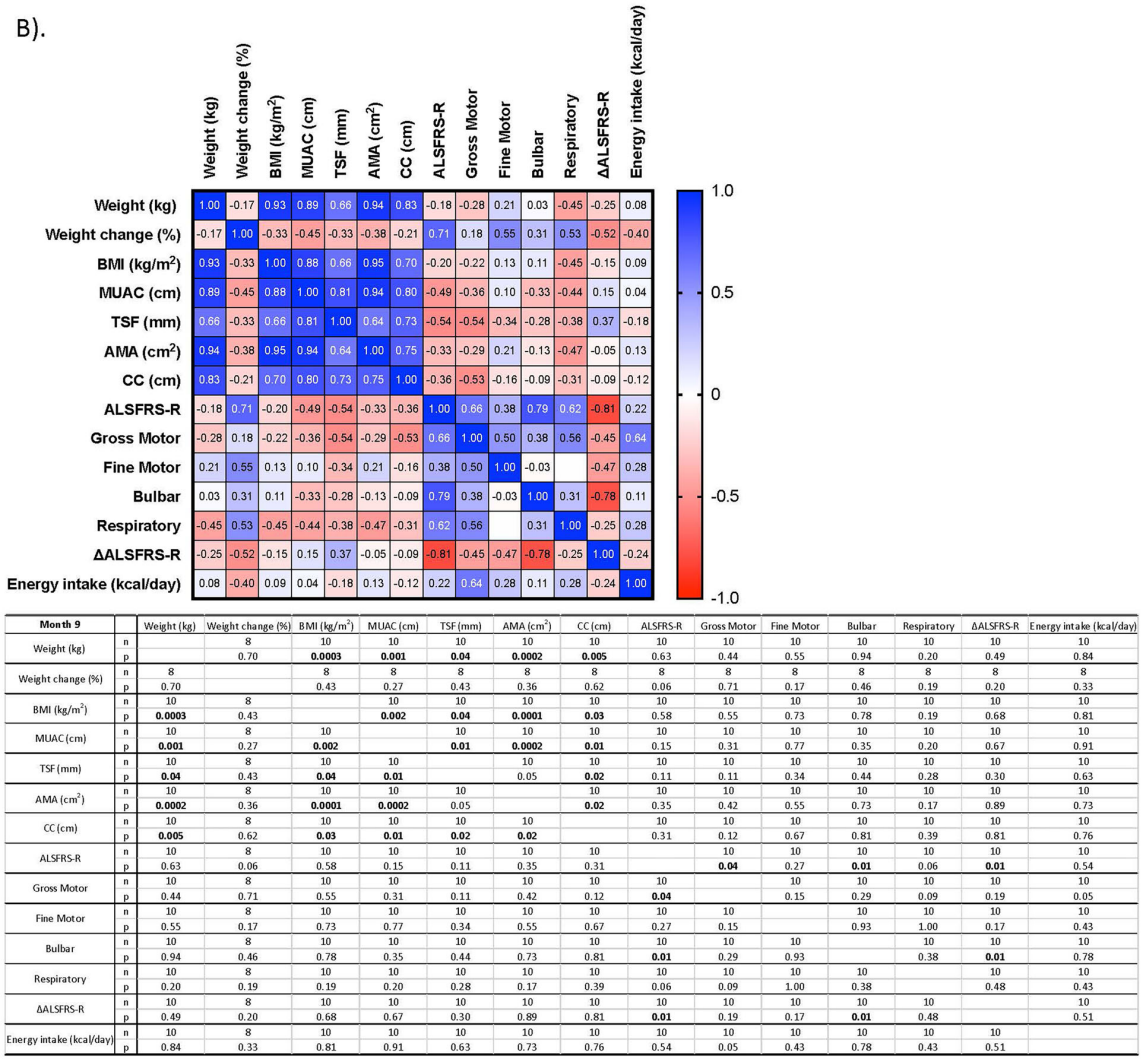

At M0, significant negative correlations were observed between the ALSFRS-R total score against weight, MUAC, TSF and CC (Figure 1A). With the exception of ALSFRS-R against TSF at M3 (data not shown), the negative relationship between disease severity and nutritional status became non-significant throughout the study period (Figure 1B). There was a significant negative relationship between energy intake and BMI at M0 (Figure 1A); however, this was not observed longitudinally.

When the cohort was split into two groups at the median percentage of weight loss at M0 (−6.1% [IQR: −7.9 – −1.8]), no significant differences were observed for disease duration, total ALSFRS-R score, or change in functional score between the groups (data not shown). When the cohort was divided by the median ALSFRS-R score at M0 (30 [IQR: 28.8-36.3]), participants with an ALSFRS-R score ≤30 had a significantly higher weight, MUAC, TSF, CC and faster rate of functional decline, although both groups were classified as slowly-progressing (36) (Table 3). No significant correlations were observed between disease duration and TSF, MUAC, AMA or CC (data not shown).

**Table 3.** Cohort split by median ALSFRS-R value (≤ 30 and > 30) at enrollment (M0). Continuous data is presented as mean (SD). Median (IQR) is presented for heavily skewed data. Mean values compared using the Mann-Whitney U test. Significance observed at *p* < 0.05, highlighted in bold. AMA: arm muscle area; ALSFRS-R: Amyotrophic Lateral Sclerosis functional rating scale – revised; BMI: body mass index; CC: calf circumference; IQR: inter-quartile range; MUAC: mid-upper arm circumference; SD: standard deviation; TSF: triceps skinfold thickness; ΔALSFRS-R: change in functional score.

	≤30 n/N = 10/18	>30 n/N = 8/18	*P*	U
ALSFRS-R, /48	n = 10 28.30 (2.06)	n = 8 37.13 (4.70)	**<0.0001**	0
ΔALSFRS-R	n = 10 0.96 (0.51)	n = 8 0.35 (0.23)	**0.008**	11
Gross Motor subscore, /12	n = 10 6.30 (2.71)	n = 8 8 (1.69)	0.13	23
Disease duration, months	n = 10 29.50 (22.99) 24 (13.5-36)	n = 8 56.50 (56.75) 29 (25-71.25)	0.12	22
Weight, kg	n = 9 89.23 (17.76)	n = 8 68.25 (12.15)	**0.03**	13
Percentage weight change, %	n = 8 −3.68 (9.48)	n = 7 −5.21 (4.24)	0.78	25
BMI, kg/m^2^	n = 9 28.12 (3.63)	n = 8 23.44 (4.07)	**0.07**	17
MUAC, cm	n = 10 31.57 (2.79)	n = 8 26.82 (2.66)	**0.002**	7
TSF, mm	n = 10 16.14 (6.93)	n = 8 10.66 (4.68)	**0.04**	16.5
AMA, cm^2^	n = 10 56.87 (14.83)	n = 8 44.22 (8.47)	0.12	22
CC, cm	n = 10 39.24 (3.27)	n = 8 34.69 (1.45)	**0.001**	6
Energy intake, kcal/day	n = 10 2234.69 (865.44)	n = 8 2243.97 (650.80)	0.41	30

### Indication of malnutrition

Intra-cohort indicators for the risk of malnutrition at each time point are presented in Table S2. The risk of malnutrition increased from 27.8% at M0, to 40% at M9 (Table 4). Those at risk of malnutrition at M0 were significantly lighter, with a lower BMI, MUAC and TSF (Table 5). However, no significant differences were identified for the disease duration, disease severity, rate of disease progression or daily energy intake between the two groups.

**Table 4.** Prevalence of the risk of malnutrition in the study cohort at each time point. Data is presented as the number of participants (n/N), percentage of study population (%). AMA: arm muscle area; BMI: body mass index; M0-9: Months 0-9; MUAC: mid-upper arm circumference; TSF: triceps skinfold thickness.

		Criteria and thresholds for risk of malnutrition					
Study visit	Prevalence of the risk of malnutrition	≥10 % weight loss	BMI ≤20 or ≤22 kg/m^2^	MUAC, cm	TSF, mm	Calf Circumference, cm	Energy intake (kcal/day)
M0	5/18 (27.8)	1/18 (5.6)	2/18 (11.1)	5/18 (27.8)	2/18 (11.1)	2/18 (11.1)	12/18 (66.7)
M3	6/16 (37.5)	3/16 (18.8)	2/16 (12.5)	5/16 (31.3)	2/16 (12.5)	1/16 (6.3)	11/16 (68.8)
M6	4/13 (30.8)	3/13 (23.1)	2/13 (15.4)	5/13 (38.5)	2/13 (15.4)	1/13 (7.7)	5/13 (38.5)
M9	4/10 (40)	3/10 (30)	2/10 (20)	4/10 (40)	2/10 (20)	1/10 (10)	6/10 (60)

**Table 5.** Comparison of clinical and nutritional assessment parameters by risk of malnutrition at enrollment (M0). Continuous data is presented as mean (SD). Median (IQR) is presented for heavily skewed data. Mean values compared using the Mann-Whitney U test. Significance observed at *p* < 0.05. AMA: arm muscle area; ALSFRS-R: Amyotrophic Lateral Sclerosis functional rating scale – revised; BMI: body mass index; CC: calf circumference; IQR: inter-quartile range; MUAC: mid-upper arm circumference; SD: standard deviation; TSF: triceps skinfold thickness; ΔALSFRS-R: change in functional score.

	No risk of malnutrition (n/N = 13/18)	Risk of malnutrition (n/N = 5/18)	*P*	*U*
Sex, Male:Female	11:2	5:0		
Age, years	64.08 (8.86)	56.80 (14.41)	0.46	24.5
ALSFRS-R, /48	31.15 (5.77)	35.00 (4.64)	0.16	18
ΔALSFRS-R	0.78 (0.57)	0.45 (0.12)	0.28	21
Disease duration, months	46.15 (49.29) 25.00 (15.50-68.50)	29.40 (9.66) 28.00 (22.00-37.50)	0.79	29.5
Weight, kg	85.03 (17.91)	65.76 (12.06)	**0.03**	10
Percentage weight change, %	−3.75 (8.49)	−6.17 (1.58)	0.85	20
BMI, kg/m^2^	27.78 (3.44)	21.46 (3.28)	**0.005**	4.5
MUAC, cm	31.11 (2.54)	25.16 (1.89)	**0.0002**	0
TSF, mm	16.49 (5.17)	9.28 (5.13)	**0.045**	11
AMA, cm^2^	53.72 (11.42)	39.66 (7.30)	0.06	12
CC, cm	37.92 (3.63)	35.39 (2.32)	0.17	18
Energy intake, kcal/day	2235.48 (860.19)	2247.45 (454.57)	0.50	25

### Comparison of anthropometric measures against estimates of body composition

Anthropometric measures and indices were correlated against estimates of body composition (FM and LBM, kg) calculated using the Tandan equation (40). Significant, positive correlations were observed between AMA and LBM (r = 0.55, *p* = 0.02, *n* = 17), as well as between TSF and FM (r = 0.69, *p* = 0.002, *n* = 17).

### Longitudinal analysis of nutritional status and disease progression

Assessments of disease progression, anthropometric measures and body composition were collected at all time points. Ten participants completed all study time points (from M0 to M9). For these participants, we observed changes of nutritional status and examined its relation to disease progression (Table 6). The ALSFRS-R total, fine- and gross-motor subscale scores significantly decreased over nine months. No significant changes were observed for any anthropometric assessment or estimate of body composition.

**Table 6.** Changes in disease progression, anthropometric measures and body composition (*n* = 10). Data presented as mean (SD). Mean values for M0 and M9 compared using paired samples T-tests or Wilcoxon tests for non-parametric data. Significance observed at *p* < 0.05, highlighted in bold.

	M0	M9	Difference (M9-M0)	*P* value
ALSFRS-R	32.30 (5.12)	26.00 (9.20)	−6.30 (6.00)	**0.009**
Bulbar subscale	9.80 (2.74)	8.20 (4.13)	−1.60 (2.22)	0.06
Fine motor subscale	6.90 (2.85)	4.80 (3.33)	−2.10 (2.08)	**0.01**
Gross motor subscale	6.80 (1.48)	4.90 (2.13)	−1.90 (2.13)	**0.02**
Respiratory subscale	8.80 (3.39)	8.10 (3.99)	−0.70 (1.64)	0.28
ΔALSFRS-R	0.62 (0.53)	0.60 (0.45)	−0.01 (0.19)	>0.99
Weight, kg	84.84 (19.30)	83.09 (16.27)	−1.78 (4.99)	0.29
BMI, kg/m^2^	26.30 (4.73)	25.20 (4.84)	−1.10 (6.34)	0.60
TSF, mm	13.60 (7.96)	12.96 (4.80)	−0.63 (6.97)	0.78
MUAC, cm	29.16 (4.37)	28.78 (4.02)	−0.38 (1.21)	0.35
AMA, cm^2^	50.54 (16.97)	49.35 (12.01)	−1.19 (12.65)	0.77
CC, cm	38.02 (3.55)	37.99 (3.01)	−0.03 (1.09)	0.94
%FM	26.10 (9.52)	24.61 (5.47)	−1.49 (6.56)	0.49
FM, kg	23.74 (13.14)	21.00 (7.39)	−2.74 (7.43)	0.27
LBM, kg	61.10 (7.18)	62.06 (9.97)	0.96 (5.60)	0.60

**Figure 2.**
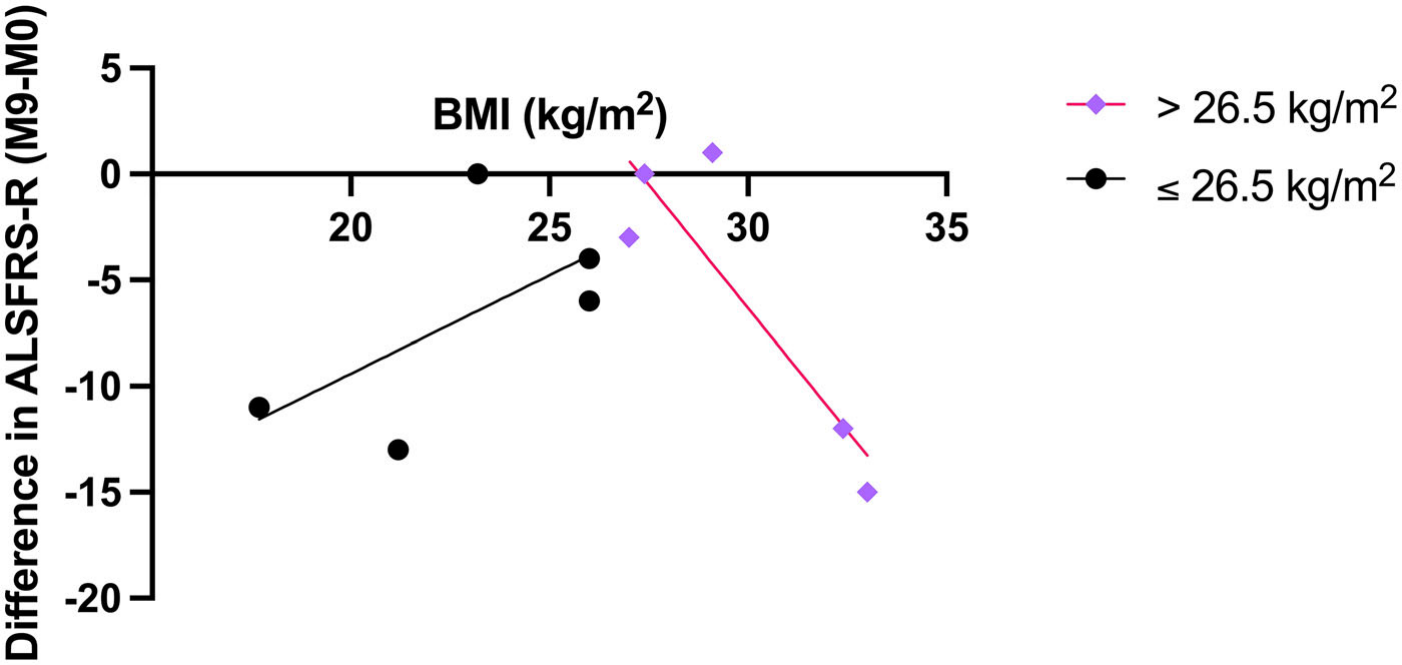
Correlation of BMI against difference in ALSFRS-R (M9-M0) (*n* = 10). Cohort divided by the median BMI value at M0 (26.5 kg/m^2^). Correlation analysis was conducted using Pearson’s correlation coefficient with significance at *p* < 0.05. BMI: body mass index.

The median BMI (26.5 kg/m^2^ [IQR: ± 22.7-29.9]) value for the 10 participants at M0 was used to classify participants into two groups. We examined the relationship between BMI and the change in ALSFRS-R total score over nine months for each group (Fig. 2). A significant, strong negative correlation was observed for those with a BMI >26.5 kg/m^2^ (r = −0.89, *p* = 0.04, *n* = 5), whilst a moderate positive relationship was observed for those with a BMI ≤26.5 kg/m^2^, but this was not significant (r = 0.62, *p* = 0.27, *n* = 5). Patients with a BMI closest to 26.5 kg/m^2^ demonstrated the smallest difference in functional score change.

**Figure 3.**
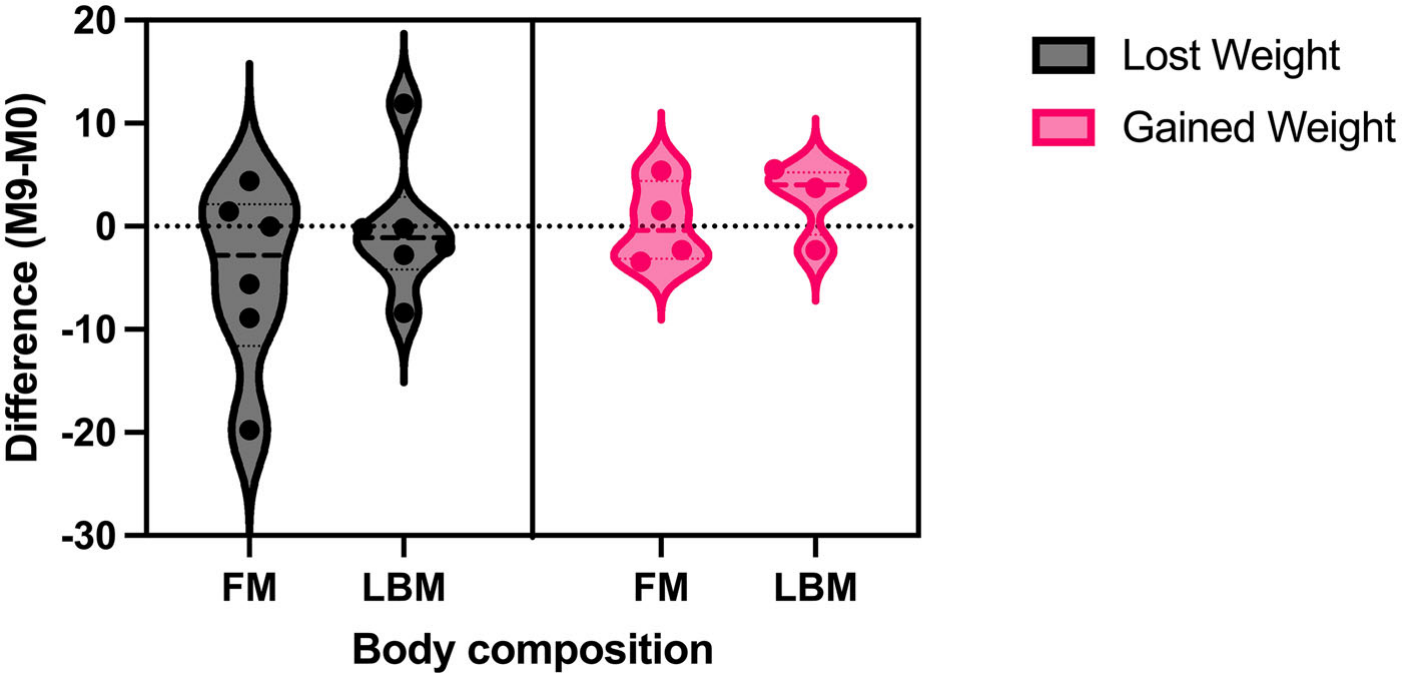
Change in body composition in participants who have lost or gained weight over the nine-month study period (Month 9 - Month 0). FM: fat mass; LBM: lean body mass; M0/9: month 0/9.

The intra-cohort percentage change for all anthropometric measurements and indices was calculated for the 10 participants between M0 and M9. High variability was observed for the percentage change in weight (range: −9%-11.3%, *n* = 10), with 60% (n/*N* = 6/10) of participants exhibiting weight loss over nine-months. In the participants who lost weight, we observed a decrease in FM in 66.7% (n/*N* = 4/6) and a decrease in LBM in 83.3% (n/*N* = 5/6). In the participants who gained weight, we observed an increase in FM in 2/4 (50%) and an increase in LBM in 3/4 (75%) (Fig. 3).

As per Tandan et al., (2022), the cohort were divided into three groups to examine the influence of body composition on clinical parameters: those who exhibited a change of > −2.5% of FM or LBM were considered to ‘lose’ FM or LBM; those who exhibited a change of ≤ ±2.5% were considered ‘stable’; and those who exhibited a change of > +2.5% were considered to ‘gain’ FM or LBM (40). Participants who lost FM (n/*N* = 5/10) exhibited a significant decrease in BMI compared to those who remained stable or gained FM. Conversely, a significant increase in BMI was observed for those who lost LBM (n/*N* = 4/10), compared to those who remained stable or gained LBM (Table 7). A significant strong positive correlation was observed between the change in body weight and the change in CC over the nine-month study period (r = 0.70, *p* = 0.02, *n* = 10).

**Table 7.** Change in clinical parameters over nine-month study period according to changes in body composition (M9-M0). Those who exhibited a change of >−2.5% of FM or LBM were considered to have a ‘reduction’ in FM or LBM; those who exhibited a change of ≤±2.5% were considered ‘stable’; and those who exhibited a change of >+2.5% were considered to have ‘gained’ FM or LBM. Data presented as mean (SD). Mean values compared using unpaired t-tests or Mann-Whitney test. Significance observed at *p* < 0.05, highlighted in bold. ALSFRS-R: amyotrophic lateral sclerosis functional rating scale – revised; BMI: body mass index; FM: fat mass; LBM: lean body mass; M0/9: month 0/9.

	Change in clinical parameters (M9-M0)		
	ALSFRS-R	Weight	BMI
**FM**			
Reduction (n = 5)	−6.40 (7.09)	−3.85 (5.35)	−5.88 (4.46)
Stable/gained (n = 5)	−6.20 (5.54)	0.29 (4.09)	3.68 (3.67)
P value	0.96	0.21	**0.008**
**LBM**			
Reduction (n = 4)	−5.00 (5.60)	−1.08 (3.11)	4.60 (3.50)
Stable/gained (n = 6)	−7.17 (6.62)	−2.24 (6.20)	−4.90 (4.66)
P value	0.61	0.74	**0.009**

## Discussion

Our intra-cohort analysis demonstrated high variability in the longitudinal changes in body mass. Nau et al., (1994) suggested that increases in FM may mask clinically-significant declines in LBM; therefore, body mass may not necessarily reflect disease progression in people with ALS, recommending that longitudinal monitoring of body composition should be considered instead (52). In agreement with Tandan et al., (2022), we observed in our study that weight loss is predominantly associated with a decline in FM and LBM (40).

We demonstrated that the anthropometric assessments of TSF and AMA in our study correlated significantly with estimates of FM and LBM (calculated using a recently developed ALS-specific equation by Tandan et al., 2022) (40), suggesting that they may be suitable surrogate indicators of body composition in this cohort. We used measurements of TSF as an estimate of subcutaneous FM and calculated AMA from measurements of TSF and MUAC to estimate LBM. The dissociation between AMA and TSF was therefore surprising, but in keeping with previously reported findings (53). MUAC and CC are unable to differentiate between FM and LBM. However, we observed a significant positive correlation between the percentage of body weight change and percentage change in CC over nine months. This suggests that changes in CC could be used as a surrogate marker to monitor weight loss in ALS. The significant, positive relationships observed between MUAC and CC against BMI in our cohort suggests that these measurements could be used as surrogate indicators of BMI; however, this requires further validation.

Our study supports the findings by Kasarskis et al. (1997) whereby AMA significantly, positively correlated with body weight and BMI in 18 people living with ALS (53). Longitudinal measurements of AMA in a subset of these participants (*n* = 10) were observed to decrease over nine months; it was therefore suggested that AMA may be used to monitor muscular atrophy in ALS (53). However, significant declines in AMA were not observed over the nine-month study period in our cohort. To consider whether this discrepancy was a result of the different equations to derive AMA available in the anthropometry literature, we calculated AMA using the Heymsfield equation^3^ (54) (referenced by Kasarskis et al., (1997)) in our cohort. We did not observe any significant longitudinal changes in AMA (data not shown). Differences in the demographics, disease severity or body composition of the two study populations may explain this discrepancy, but definite comparisons cannot be drawn as these data was not reported by Kasarskis et al., (1997) (53).

### Relationship of nutritional status assessed by body composition with function and disease progression

The ΔALSFRS-R was observed to decline at a rate of 0.3-0.7 points/month over the nine-month period demonstrating decreased function at a slow progression rate within this cohort. Despite a mean percentage of weight loss of −4.4% [SD: 7.3] at M0 (compared to initial weight), this cohort was considered to be overweight. A higher BMI is considered to be protective for survival in ALS (55). Indeed, we observed that patients with a BMI closest to 26.5 kg/m^2^ demonstrated the smallest difference in functional score between M0 and M9. The concept of a non-linear relationship between BMI and change in ALSFRS-R score in ALS has been introduced by Reich-Slotky et al., (2013) (56). The authors reported that for individuals in their cohort (*n* = 150) with a BMI of <30 kg/m^2^, a higher BMI was associated with a slower ALSFRS-R decline, whilst for individuals with a BMI of >30 kg/m^2^, a higher BMI was associated with a faster rate of functional decline.

The significant negative correlation between TSF and disease severity observed at M0 and M3 in our study reflects results published by Salvioni et al., (2015) who demonstrated significant negative correlations between TSF and ALSFRS score in a cross-sectional study of 111 people living with ALS (57). It would have been interesting to observe survival analysis data from our cohort to contribute toward the hypothesis that a higher FM is protective in ALS (10).

### Indication of malnutrition

We used anthropometric assessments and participant-reported energy intake to identify individuals at risk of developing malnutrition in our cohort. Longitudinal dietary intake analysis for our cohort showed that approximately 40-70% of our cohort did not consume the recommended daily energy intake according to age and sex at different time points across this study (Table S2F). This agrees with dietary analysis published from other ALS populations (9,19,58,59). The lack of standardized guidelines for identifying malnutrition in ALS can result in different estimates of its prevalence. For example, using the two-criteria approach outlined in Table 1, 27.8% of our cohort were identified as being at risk of malnutrition at M0. Conversely, had we applied a threshold of ≥ 5% weight loss without further criteria, 50% of this cohort would have been indicated as being at risk of malnutrition at M0.

Measurements of MUAC, TSF and AMA below the 5^th^ percentile of anthropometry reference databases (60,61) were previously used to identify malnutrition in an independent ALS cohort (19). However, these databases were developed in the 1980s and may not reflect the body composition of today’s population. Measurements of MUAC, CC and TSF in our study were therefore compared to percentiles published in the National Health and Nutrition Examination Surveys (NHANES)^4^ (2003–2018) for age and sex (62). To our knowledge, these are the most recent, open-access databases to which our data could be compared; no open-access databases exist for anthropometric data in ALS. This limits direct comparisons to other ALS cohorts and potentially biases toward an inappropriate classification of nutritional status. We call for greater transparency when collecting and reporting anthropometric assessments across ALS cohorts. The provision of data sharing could enable the creation of a comprehensive, international database which could be used to critically examine changes in body composition with disease stratification.

### Considerations

This study presents anthropometric data from a small number of patients living with typically-progressing ALS. Compared to a representative ALS population at diagnosis in the UK (63), our predominantly-male, White British cohort was younger and lighter (64). Recruitment was not restricted by disease severity or nutritional status: on average, this cohort presented with moderate disease severity, with a higher ALSFRS-R score compared to other nutritional and metabolic studies in ALS (65–69). The median disease duration of 25.5 months [IQR: 18.5-49.5] and the average change in ALSFRS-R score (0.7 [SD: 0.5]) indicates a potential bias toward the inclusion of slower-progressing individuals (70). For these reasons, the relationship between function and anthropometric assessments observed in this cohort may have a low external validity and these results may not be necessarily applicable to another independent ALS cohort with different characteristics.

#### Quality control

Anthropometric indices and thresholds are based on underlying theoretical assumptions linked to the body composition, age and sex of the cohort from which they were derived (71); the accuracy of these indices is therefore population-dependent. The accuracy and reliability of anthropometric assessments in a cohort experiencing lower-than-predicted LBM, such as in ALS, may therefore be reduced (72).

The body composition of an individual with ALS constantly changes throughout disease progression. Reliable anthropometric measurements assume that the point of measurement is representative of whole-body composition and symmetry of limb muscles. A single TSF or circumference measurement may therefore be insufficient to detail these changes. Gross or observational error when recording symmetrical TSF measurements was mitigated by assessing intra-evaluator reliability by calculating the relative TEM (73).

Although Intake24 has been established for its convergent- and criterion- validity (74–79), all intake data reported in this manuscript are participant-reported estimates and did not undergo verification at the time of data collection. We therefore acknowledge the potential for error in the estimations of portion sizes or energy intake, for example.

#### Missing data

It was not possible to calculate the percentage of weight change where: i) initial body weight was not provided (*n* = 2); or ii) current body weight measurements were not collected in non-ambulatory individuals (M0: *n* = 1). Restrictions in limb movement removed the ability to measure/derive MUAC, TSF, AMA and CC (M6: *n* = 1).

## Conclusion

We have shown that limb anthropometric assessments correlated with weight, BMI and disease severity in this study cohort. These assessments may therefore act as surrogate indicators of nutritional status. However, these measurements provide localized, crude estimates of body composition, and the inter-evaluator reliability and robustness of these measurements need further validation in a larger study population. Further investigation is required by means of large-scale, multi-centre studies to collect body composition data and construct open-access ALS databases to develop clinically-meaningful thresholds and percentiles.

## Supplementary Material

Supplementary material_v2_CLEAN.docx

## Footnotes

1 % body fat = 1.73 − (19.80 x ‘gender’) + (0.25 x weight, kg) + (0.952 x BMI, kg/m^2^) – (5.20 x ‘onset’). Where gender = 1 if male, 0 if female; and bulbar-onset = 1, limb-onset = 0.

2 FM (kg) = %FM x (body weight/100); LBM (kg) = body weight – FM (kg).

3 AMA (cm^2^) (males) = [MUAC (cm) – (TSF (mm) x π)]^2/^(4 x π) – 10; AMA (cm^2^); (females) = [MUAC (cm) – (TSF (mm) x π)]^2/^(4 x π) – 6.5 (50).

4 A series of annual studies to examine the nutritional status of a representative sample of 5,000 adults and children in the U.S (58).

## Rights retention statement

For the purpose of open access, the author has applied a Creative Commons Attribution (CC BY) license to any Author Accepted Manuscript version arising from this submission.
