# Supplementary material for: Exploring the role of anthropometric measurements to assess nutritional status in amyotrophic lateral sclerosis: a longitudinal prospective cohort study
Source: Amyotroph Lateral Scler Frontotemporal Degener. 2024 Dec 16;26(3-4):225–38. doi: 10.1080/21678421.2024.2434176 (PMC12011022; doi:10.1080/21678421.2024.2434176)
Supplement: Supplementary material_v2_CLEAN.docx [file IAFD_A_2434176_SM8987.docx]

Supplementary Table 1. Difference between left and right measurements for MUAC, TSF, AMA and calf circumference at all time points.

Statistical analysis was performed using a paired samples t test. Significance was determined at the p < 0.05 level. AMA: arm muscle area; MUAC: mid upper arm circumference; TSF: Triceps skinfold thickness.

|  | **MUAC** | **TSF** | **AMA** | **CC** |
| --- | --- | --- | --- | --- |
| **M0** | 0.65 | 0.42 | 0.39 | 0.67 |
| **M3** | 0.14 | 0.73 | 0.48 | 0.33 |
| **M6** | 0.83 | 0.30 | 0.64 | 0.38 |
| **M9** | 0.37 | 0.74 | 0.70 | 0.30 |

Supplementary Table 2. Longitudinal indicators for the risk of malnutrition by (A) percentage weight change from initial body weight (%), (B) BMI (kg/m^2^), (C) MUAC (cm), (D) calf circumference (cm), (E) TSF (mm) and (F) Estimated energy intake (kcal/day).

Values VaalmdaValues below each threshold for malnutrition are highlighted in bold. Data not collected is denoted by ‘-‘. BMI: body mass index; CC: calf circumference; MUAC: mid upper arm circumference; TSF: triceps skinfold thickness.

1. **Percentage weight change (%)**

|  | **M0** | **M3** | **M6** | **M9** |
| --- | --- | --- | --- | --- |
| Pt01 | -4.81 | -5.56 | 2.22 | 5.93 |
| Pt03 | -9.76 | -8.28 | **-11.35** | **-15.81** |
| Pt04 | -6.07 | -3.94 | - | **-** |
| Pt06 | **-16.54** | **-10** | - | - |
| Pt07 | 0.81 | 2.56 | 0 | 1.16 |
| Pt09 | - | **-** | - | **-** |
| Pt10 | -7.09 | **-16.39** | **-** | - |
| Pt11 | -8.57 | **-** | **-** | - |
| Pt13 | -7.89 | -8.68 | -8.68 | -8.68 |
| Pt14 | 15.95 | 14.39 | 19.14 | 9.37 |
| Pt15 | - | - | - |  |
| Pt16 | -6.39 | -8.43 | **-12.22** | **-14.81** |
| Pt17 | - | - | - |  |
| Pt19 | -7.14 | **-10** | **-14.57** | **-12.86** |
| Pt20 | 3.85 | 1.65 | 5.63 | 7.57 |
| Pt21 | -1.76 | -3.53 | -3.53 |  |
| Pt23 | -4.84 | -2.26 | -0.97 |  |
| Pt24 | -5.69 | -2.44 | -2.33 |  |

1. **BMI (kg/m^2^)**

|  | **M0** | **M3** | **M6** | **M9** |
| --- | --- | --- | --- | --- |
| Pt01 | **17.7** | **17.6** | **19** | **19.7** |
| Pt03 | 32.4 | 32.9 | 31.7 | 30.2 |
| Pt04 | 26.4 | 27 | - | - |
| Pt06 | 22.2 | 23.9 | - | - |
| Pt07 | 26 | 26.3 | 25.7 | 26 |
| Pt09 | 27.4 | 27.9 | 27.8 | 28.3 |
| Pt10 | 29 | 26.1 | - | - |
| Pt11 | 22.8 | - | - | - |
| Pt13 | 23.2 | 23 | 23 | 23 |
| Pt14 | 29.1 | 28.7 | 29.9 | 27.4 |
| Pt15 | 26 | 25.4 | 26.2 | 25.2 |
| Pt16 | 33 | 32.3 | 30.9 | 30 |
| Pt17 | - | - | - | - |
| Pt19 | 21.2 | 20.5 | **19.5** | **19.9** |
| Pt20 | 27 | 26.4 | 25.6 | 28 |
| Pt21 | 26.4 | 25.9 | 26 | - |
| Pt23 | **19.2** | **19.7** | 20.05 | - |
| Pt24 | 31.6 | 32.7 | 32.83 | - |

**(C) MUAC (cm)**

Values Vaalmda

|  | **M0** | **M3** | **M6** | **M9** |
| --- | --- | --- | --- | --- |
| Pt01 | **22.25** | **21.7** | **21.9** | **22.75** |
| Pt03 | 37.00 | 35.05 | 34.45 | 34.25 |
| Pt04 | 30.90 | 29.75 | - | - |
| Pt06 | 28.50 | 27.25 | - | - |
| Pt07 | 32.35 | 31.2 | 31.55 | 31.3 |
| Pt09 | 30.15 | 29.75 | 30.7 | 29.75 |
| Pt10 | 27.35 | 26.2 | - | - |
| Pt11 | 28.55 | - | - | - |
| Pt13 | **24.25** | **23.75** | **23.8** | **23.35** |
| Pt14 | 31.00 | 31.3 | 32.4 | 30.95 |
| Pt15 | **26.75** | **28.65** | **27.6** | **27.9** |
| Pt16 | 32.20 | 32.9 | 32.7 | 33.15 |
| Pt17 | 31.15 | - | - | - |
| Pt19 | **26.20** | **25.7** | **24.7** | **24.65** |
| Pt20 | 29.45 | 28.55 | 28.45 | 29.75 |
| Pt21 | 31.80 | 30.75 | - | - |
| Pt23 | **26.35** | **25.25** | **26.4** | - |
| Pt24 | 34.00 | 33 | 33.65 | - |

**(D) Calf circumference (cm)**

|  | **M0** | **M3** | **M6** | **M9** |
| --- | --- | --- | --- | --- |
| Pt01 | **32.75** | **33.05** | **33.75** | **33.35** |
| Pt03 | 44.9 | 44.75 | 44.15 | 43.45 |
| Pt04 | 38.7 | 38.35 | - | - |
| Pt06 | 33.3 | 32.95 | - | - |
| Pt07 | 39.35 | 38.5 | 39.3 | 38.65 |
| Pt09 | 36.45 | 36.7 | 36.9 | 37.35 |
| Pt10 | 34.65 | 33.95 | - | - |
| Pt11 | **32.45** | - | - | - |
| Pt13 | 34.85 | 34.8 | 34.6 | 35 |
| Pt14 | 41.2 | 41.25 | 40.95 | 41.05 |
| Pt15 | 39.1 | 39.8 | 38.15 | 38.85 |
| Pt16 | 39.85 | 39.25 | 38.75 | 38.8 |
| Pt17 | 36.5 | - | - | - |
| Pt19 | 35.55 | 34.95 | 34.25 | 34.95 |
| Pt20 | 36.15 | 37.65 | 38.15 | 38.45 |
| Pt21 | 36.9 | 36.3 | - | - |
| Pt23 | 34.7 | 34.3 | 34.65 | - |
| Pt24 | 42.6 | 42.65 | 42.75 | - |

**(E) TSF (mm)**

|  | **M0** | **M3** | **M6** | **M9** |
| --- | --- | --- | --- | --- |
| Pt01 | 6.6 | 8.6 | 7.2 | 10.9 |
| Pt03 | 17.6 | 20.7 | 19.0 | 19.9 |
| Pt04 | 9.0 | 9.2 | - | - |
| Pt06 | 16.2 | 18.2 | - | - |
| Pt07 | - | 18.8 | 18.3 | 16.7 |
| Pt09 | 9.0 | 9.0 | 9.1 | **6.5** |
| Pt10 | 16.0 | 12.0 | - | - |
| Pt11 | 14.3 | - | - | - |
| Pt13 | 10.4 | 11.3 | 10.7 | 10.8 |
| Pt14 | 22.6 | 14.1 | 14.9 | 13.3 |
| Pt15 | 17.8 | 12.1 | 13.1 | 12.6 |
| Pt16 | 26.7 | 20.8 | 17.2 | 17.2 |
| Pt17 | 17.4 | - | - | - |
| Pt19 | **5.7** | **5.8** | **5.4** | **5.1** |
| Pt20 | 17.4 | 13.9 | 15.7 | 16.6 |
| Pt21 | 11.9 | - | - | - |
| Pt23 | **6.0** | **5.3** | **5.7** | - |
| Pt24 | 20.0 | 16.1 | 22.1 | - |

**(F) Participant-reported energy intake (kcal/day) in relation to daily recommended intake values according to age and sex.**

**
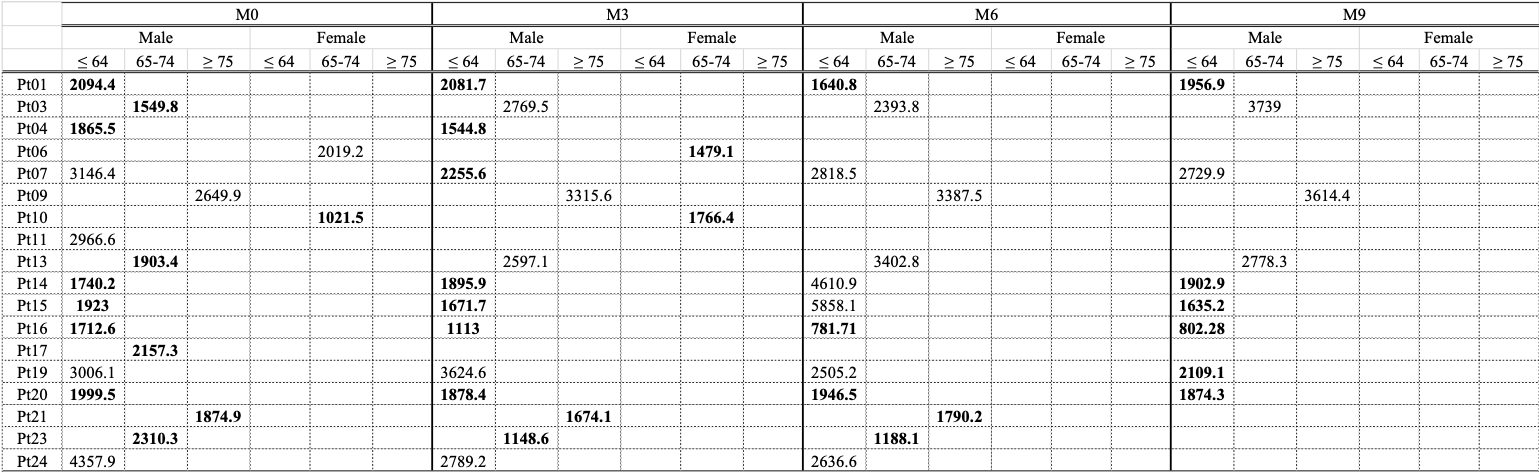
**
